# A comprehensive analysis of the role of QPRT in breast cancer

**DOI:** 10.1038/s41598-023-42566-4

**Published:** 2023-09-18

**Authors:** Yiqing Yan, Lun Li, Zixin Wang, Jian Pang, Xinyu Guan, Yunchang Yuan, Zhenkun Xia, Wenjun Yi

**Affiliations:** 1 Department of General Surgery, The Second Xiangya Hospital, Central South University, No. 139, Renmin Central Road, Changsha, 410011 China; 2 Department of Thoracic Surgery, The Second Xiangya Hospital, Central South University, No. 139, Renmin Central Road, Changsha, 410011 China

**Keywords:** Breast cancer, Tumour biomarkers, Tumour immunology

## Abstract

To explore the clinical role of QPRT in breast cancer. The gene expression, methylation levels and prognostic value of QPRT in breast cancer was analyzed using TCGA data. Validation was performed using the data from GEO dataset and TNMPLOT database. Meta analysis method was used to pool the survival data for QPRT. The predictive values of QPRT for different drugs were retrieved from the ROC plot. The expression differences of QPRT in acquired drug-resistant and sensitive cell lines were analyzed using GEO datasets. GO and KEGG enrichment analysis were conducted for those genes which were highly co-expressed with QPRT in tissue based on TCGA data and which changed after QPRT knockdown. Timer2.0 was utilized to explore the correlation between QPRT and immune cells infiltration, and the Human Protein Atlas was used to analyse QPRT’s single-cell sequencing data across different human tissues. The expression of QPRT in different types of macrophages, and the expression of QPRT were analysed after coculturing HER2+ breast cancer cells with macrophages. Additionally, TargetScan, Comparative Toxicogenomics and the connectivity map were used to research miRNAs and drugs that could regulate QPRT expression. Cytoscape was used to map the interaction networks between QPRT and other proteins. QPRT was highly expressed in breast cancer tissue and highly expressed in HER2+ breast cancer patients (*P* < 0.01). High QPRT expression levels were associated with worse OS, DMFS, and RFS (*P* < 0.01). Two sites (cg02640602 and cg06453916) were found to be potential regulators of breast cancer (*P* < 0.01). QPRT might predict survival benefits in breast cancer patients who received taxane or anthracycline. QPRT was associated with tumour immunity, especially in macrophages. QPRT may influence the occurrence and progression of breast cancer through the PI3K-AKT signalling pathway, Wnt signalling pathway, and cell cycle-related molecules.

## Introduction

Data from the International Agency for Research on Cancer (IARC) show that breast cancer has become the highest incidence of malignant tumours in the world and one of the leading causes of death in women^1^. Breast cancer is classified into different biological subtypes with complex heterogeneity. In clinical practice, there are four common molecular types, including luminal-A, luminal-B, human epidermal growth factor receptor 2 (HER2)-positive and triple-negative^2^, leading to various treatments^3^. Although breast cancer is one of the solid tumours with the best treatment effects, the heterogeneity of different breast cancer molecular subtypes leads to different treatments^4^, making it difficult to accomplish personalized precision therapy in patients.

Despite great progress in present frontal and effective treatments, systemic therapy still cannot completely cure metastatic breast cancer to improve quality of life^5^, alleviate symptoms caused by metastasis, and prolong relative long-term survival^6^. Considering the significant mortality rate, it is essential to explore new therapies for inhibiting the development of malignant systemic metastasis. The biggest challenge in the treatment of metastatic breast cancer is resistance to systemic chemotherapy and targeted therapy, which has been insurmountable thus far. Metastatic breast cancer must be treated on an individualized basis. Clinically, combined chemotherapy can improve the drug response rate and prolong progression-free survival in some patients. However, the gradual formation of drug resistance causes drug therapy to lose considerable clinical benefits. There are many known mechanisms of drug resistance, including accelerating drug efflux, drug activation and inactivation, drug target changes, and epigenetic modifications produced or obtained by mutations^7^. Several strategies have been designed to prevent or overcome resistance to systemic anticancer therapy, including drug combinations and sequential regimens. However, it seems that resistance to existing drugs and treatment regimens is inevitable in patients^8^. Therefore, we hope to further clarify the cellular and molecular processes of drug resistance in tumour cells, use new drugs to combat these mechanisms, and gradually improve the prognosis of patients with metastatic breast cancer^9^.

Quinolinate phosphoribosyltransferase (QPRT) is a key enzyme for tryptophan degradation to nicotinamide adenine dinucleotide (NAD+), collectively known as the kynurenine pathway. NAD+ is a coenzyme of many dehydrogenases in the human body, which can transfer electrons and connect the tricarboxylic acid cycle and respiratory chain. The function of NAD+ is to transfer the removed hydrogenic ionization to flavoprotein during the metabolic process. Ullmark et al. found that overexpression of QPRT in K562 cells increased drug resistance to imatinib and suggested that QPRT is a new direct target gene of WT1 in leukaemia cells^10^. Moreover, the overexpression of QPRT promoted the growth, migration, and invasion of estrogen receptor (ER)-positive BC cells^11^.

Therefore, in this study, QPRT was studied in terms of its expression, regulation, and interaction as well as its effect on prognosis in BC tissue by TCGA data, which provided an experimental basis for further exploring the molecular mechanisms of QPRT and a reference for clinical diagnosis, treatment and prognosis in BC.

## Materials and methods

### The expression data of QPRT and gene correlation analysis

Expression data of the QPRT gene were obtained from several databases, including the UALCAN database^12^, bc-GenExMiner database^13^, TNMPLOT database^14^, and multiple breast cancer-related GEO datasets. Differential expression analysis of QPRT in different clinical and pathological factors, such as tumour stage, breast cancer subgroups, lymph node metastasis, and TP53 mutations, was performed using the UALCAN online database and bc-GenExMiner database. The relationships between QPRT expression levels and overall survival (OS), distant metastasis-free survival (DMFS), and relapse-free survival (RFS) were explored by Kaplan‒Meier plotter^15^ and bc-GenExMiner. The data from multiple datasets were combined using a meta-analysis approach to analyse the relationship between QPRT expression levels and survival.

Using the linkedomics database^16^, GSE151521 dataset^17^, and Cytoscape, QPRT-related genes were selected. Subsequently, the DAVID online analysis tool^18^ and KOBAS^19^ were utilized to perform GO and KEGG enrichment analysis^20^ on these genes, aiming to elucidate the potential signalling pathways and biological processes that may be affected.

### Analysis of QPRT methylation levels

Based on TCGA (The Cancer Genome Atlas) data on the MethSurv^21^ online database and Wanderer^22^, the relationship among methylation sites, QPRT expression, and survival analysis was performed between breast cancer tissue and normal tissue.

### Predictive values of QPRT in cancer treatments

According to ROCplot^23^, the predictive values of QPRT in different breast cancer treatments were obtained. The final outcomes with RFS at 5 years and pathological complete response (pCR) in different therapies were able to predict the breast cancer patients’ survival values.

### QPRT expression in tumour-acquired drug resistance

By downloading data from multiple GEO datasets of acquired drug resistance in tumours, including GSE143944, GSE130437, GSE98987, GSE130437, GSE67916, GSE76540, GSE15043, GSE90564, GSE99225, the expression differences of QPRT between drug-sensitive and drug-resistant groups were analyzed.

### QPRT single-cell analyses and immune infiltrate abundances in breast cancer

The Human Protein Atlas (HPA)^24^ was used to explore QPRT expression in different human tissues by single-cell expression correlation analysis. TIMER2.0^25^ was used to explore the association between immune infiltrates and QPRT expression in TCGA.

### Cell culture and real-time quantitative PCR (qPCR)

BT-474, SK-BR-3, and THP-1 cells were obtained from Procell with the passing identification of short tandem repeats and qPCR detection of Mycoplasma. The culture media for the BT-474 cell line contained Roswell Park Memorial Institute (RPMI)-1640, 10 μg/ml insulin, 20% foetal calf serum (FBS), and 1% penicillin–streptomycin (P/S). The culture media conditions for the SK-BR-3 cell line contained McCoy’s 5A, 10% FBS, and 1% P/S. The culture media for the THP-1 cell line contained RPMI-1640, 10% FBS, 0.05 mM β-mercaptoethanol, and 1% P/S. THP-1 monocytes were induced to differentiate into macrophages by adding PMA (100 ng/ml) to the culture medium for 48 h. Then, macrophages were stimulated with LPS (100 ng/ml) and IFN-γ (20 ng/ml) for 48 h to induce M1 macrophage polarization. The addition of IL-4 (20 ng/ml) and IL-13 (20 ng/ml) for 48 h induced macrophage polarization to M2 macrophages.

Total RNA was extracted by TRIzol reagent (Invitrogen, 15596026) and reverse transcribed by HiScript II Reverse Transcriptase (Vazyme, R212-01). Then, 2X Universal SYBR Green Fast qPCR Mix (ABclonal, RK21203) was used for qPCR detection.

Gene primer sequences used in this study were as follows: QPRT Forwards: 5′-GCTGGTGCCGACCTTGTCCT-3′; Reverse: 5′-TCCACAGCCACACTCGGGAAC-3′; GAPDH Forwards: 5′-AACGGGAAGCTTGTCATCAA-3′; Reverse: 5′-TGGACTCCACGACGTACTCA-3′.

### Expression targeted regulation of QPRT

The miRNAs that regulate QPRT expression were predicted using the TargetScan online database^26^. Based on Cytoscape, the genes or protein molecules interacting with QPRT were selected, and a protein–protein interaction (PPI) network was constructed. The Comparative Toxicogenomics Database^27^ and the connectivity map^28^ were used to identify targeted drugs that affect pathogenesis and tumour progression in breast cancer.

### Statistical analysis

Experimental data are presented as the mean ± 95% CI. and were analysed using GraphPad Prism 9.0 software. Two-tailed unpaired Student’s t-test was used to calculate *P* values. A two-tailed *P* value of 0.05 was considered statistically significant. The expression data of QPRT were retrieved from GEO datasets, and its prognostic values in terms of overall survival, recurrence-free survival, DMFS, and disease-free survival were analysed using SPSS software. Data from several datasets for the same outcomes were pooled using Revman Software. The fixed effect model was used if the heterogeneity was small (I^2^ < 40%); otherwise, the random effect model was used.

### Patient and public involvement statement

Patients or the public were not involved in the design, conduct, reporting, or dissemination plans of our research.

## Results

### The expression and characteristics of QPRT in breast cancer tissue

**Figure 1:**
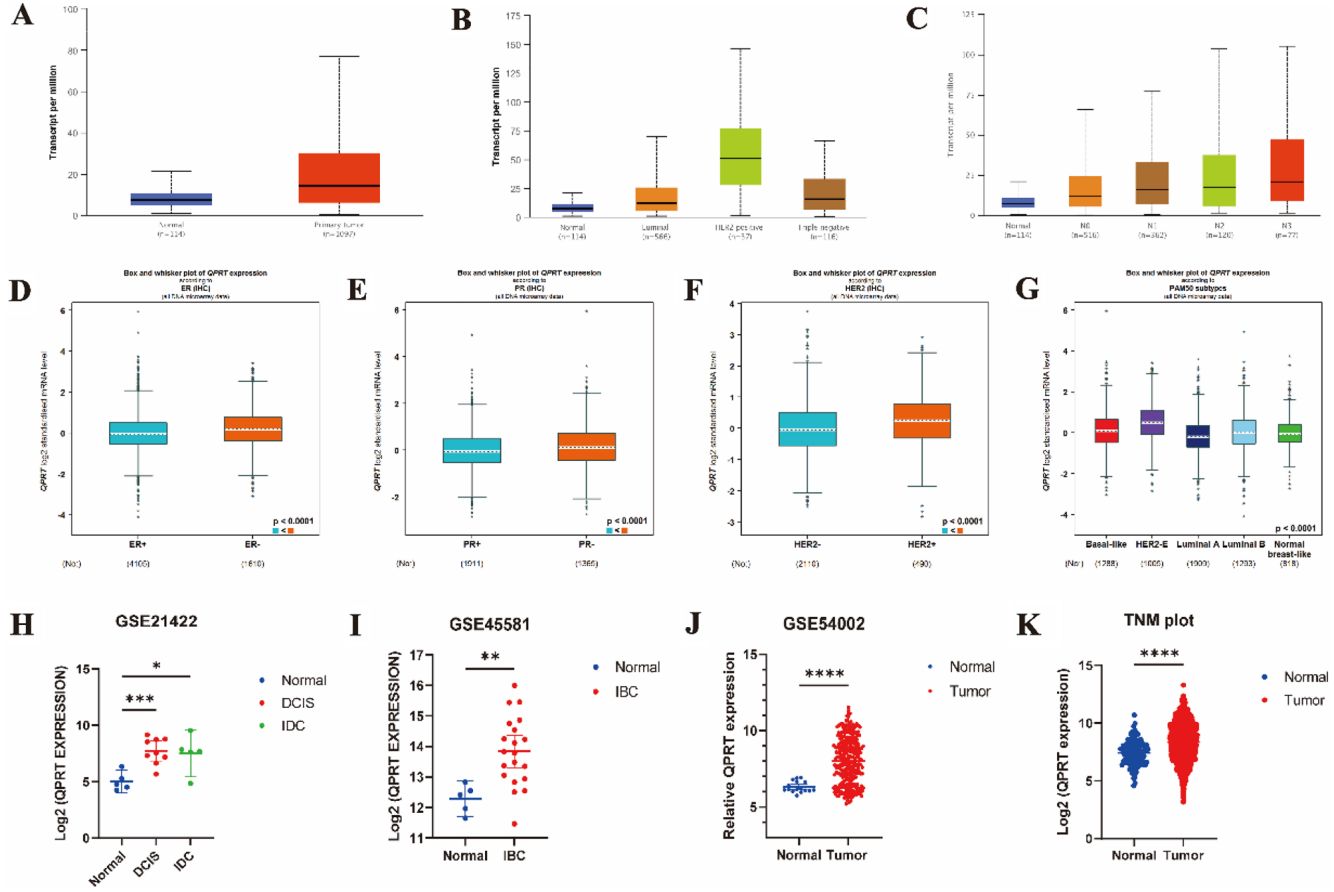
The expression and characteristics of QPRT in breast cancer tissue. (**A**) The QPRT expression differences between normal and primary tumor tissues in BRCA were compared on UALCAN. (**B**) The expression of QPRT across different subclasses of breast cancer in TCGA was analyzed by the UALCAN database. (**C**) The expression of QPRT in breast cancer about different node statuses in TCGA was analyzed by the UALCAN database. (**D**) The expression of QPRT between ER+ and ER- breast cancer in GEO using the bc-GenExMiner database. (**E**) The expression of QPRT between PR+ and PR− breast cancer in GEO using the bc-GenExMiner database. (**F**) The expression of QPRT between HER2+ and HER2- breast cancer in GEO using the bc-GenExMiner database. (**G**) The expression of QPRT across different PAM50 subtypes of breast cancer in GEO using the bc-GenExMiner database. (**H**) Log2 gene expression of QPRT gene in DCIS and IDS patients compared to healthy samples in GSE21422. (**I**) Log2 gene expression of QPRT gene in IBC patients compared to healthy samples in GSE45581. (**J**) Gene expression of QPRT gene in patients with breast cancer compared to healthy samples in GSE54002. (**K**) Log2 gene expression of QPRT gene in BRCA patients compared to healthy samples in TNM plot. Two-tailed unpaired Student’s t-test was used to calculate *P* values. (**H**)–(**K**) Data are presented as the mean ± 95%CI. * *P* < 0.05; ** *P* < 0.01; *** *P* < 0.001, ****P < 0.0001. BRCA: Breast invasive carcinoma, CI: Confidence interval, DCIS: The ductal carcinoma in situ, IBC: Inflammatory breast cancer, IDS: Invasive ductal carcinoma.

Based on The Cancer Genome Atlas (TCGA) datasets, the expression of QPRT was significantly higher in BC tissue than in normal breast tissue (*P* < 0.0001) (Fig. 1A–B). In terms of clinical characteristics, high expression of QPRT was correlated with lymph node metastasis (all *P* < 0.05) (Fig. 1C), and high QPRT expression levels were associated with nodal positive statuses (Fig. S1A) and mutated P53 (Fig. S1B) in the Gene Expression Omnibus (GEO) databases. As shown in GEO databases, ER-negative (ER−), progesterone receptor-negative (PR−), and HER2-positive (HER2+) breast cancer patients had high QPRT expression (*P* < 0.0001) (Fig. 1D–F). In the molecular subtyping of breast cancer, HER2+ breast cancer patients had higher QPRT expression than luminal A/B and TNBC patients (*P* < 0.01) (Fig. 1B). Furthermore, HER2+ breast cancer patients had the highest level of QPRT, which was much higher than that in luminal B breast cancer patients (*P* < 0.0001). Basal-like breast and normal breast-like cancer patients were lower than HER2+ breast cancer patients. Luminal A breast cancer patients had the lowest QPRT expression (Fig. 1G). To verify QPRT expression in BC tissue and normal breast tissue, we included different GEO gene expression datasets and TNMplot datasets in our analysis. We found that QPRT was more highly expressed in BC tissue than in normal breast tissue within these datasets (Fig. 1H–K).

### The relationship between QPRT expression and prognosis in breast cancer

**Figure 2:**
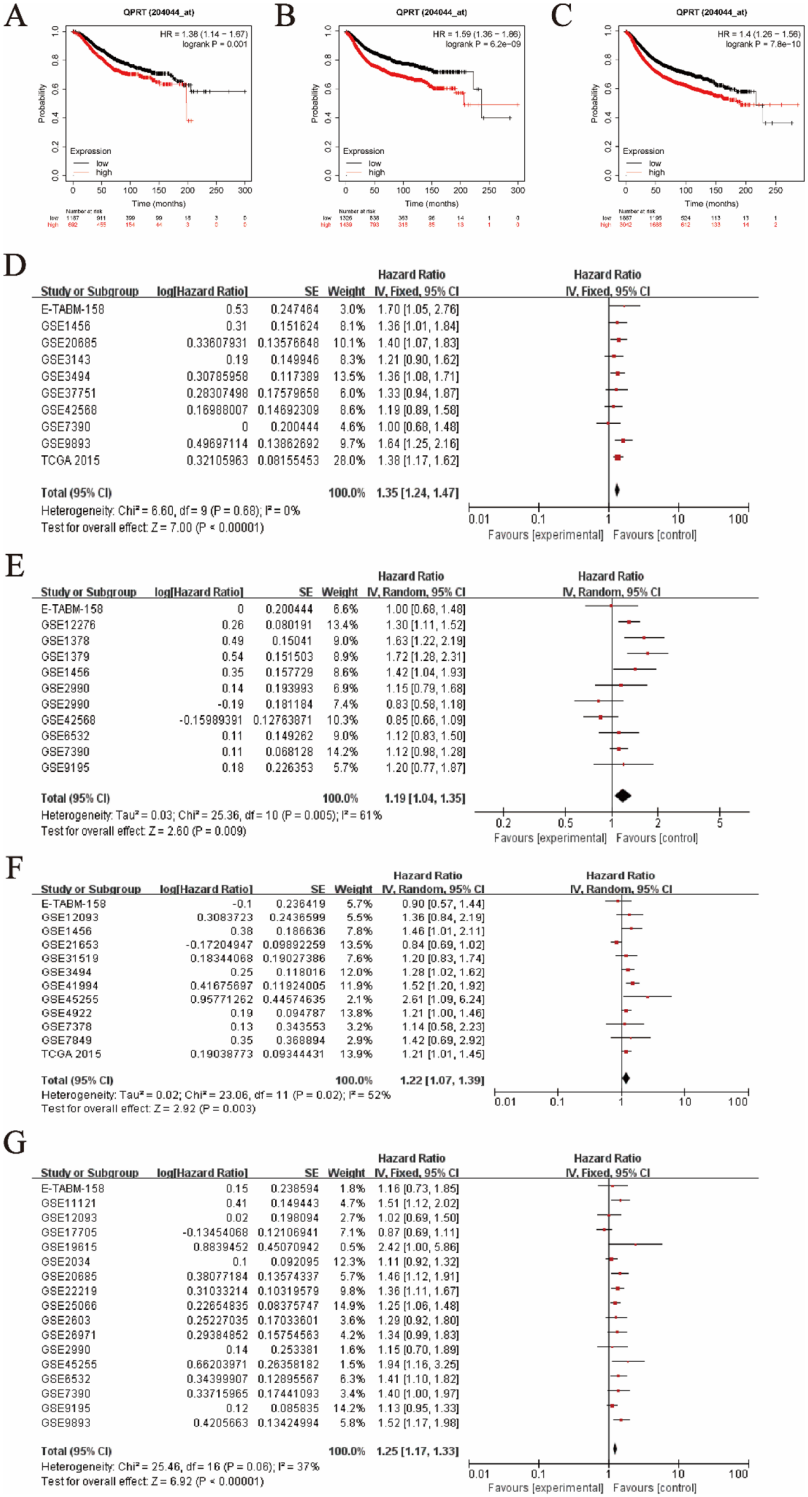
The relationship between QPRT expression and prognosis values in breast cancer. Overall survival (OS) between the high-low expression groups of QPRT gene in Kaplan–Meier Plotter. (**B**) Distant metastasis-free survival (DMFS) between the high-low expression groups of QPRT gene in Kaplan–Meier Plotter. (**C**) Relapse-free survival (RFS) between the high-low expression groups of QPRT gene in Kaplan–Meier Plotter. (**D**) Ten datasets (2822 samples) assessed the relationship between QPRT and overall survival. (**E**) Eleven datasets (2085 samples) assessed the relationship between QPRT and recurrence-free survival. (**F**) Twelve datasets (3180 samples) assessed the relationship between QPRT and disease-free survival. (**G**) Seventeen datasets (4100 samples) assessed the relationship between QPRT and distant metastasis-free survival.

The results from the Kaplan‒Meier plotter revealed that breast cancer patients with high QPRT levels had shorter overall survival (OS) (HR 1.38, 95% CI 1.14–1.67, *P* = 0.01) (Fig. 2A), distant metastasis-free survival (DMFS) (HR 1.59, 95% CI 1.36–1.86, *P* < 0.01) (Fig. 2B) and relapse-free survival (RFS) (HR 1.40, 95% CI 1.26–1.56, *P* < 0.01) (Fig. 2C). The analysis results from the bc-GenExMiner database also confirmed that patients with the high QPRT expression had worse OS, DMFS, and RFS than those with the low QPRT expression (*P* < 0.0001). The prognostic results were consistent among ER+, PR+, and HER2+ receptor statuses and different intrinsic molecular subtypes (*P* < 0.05) (Fig. S2).

In addition, we used meta-analysis methods to pool data from multiple datasets to analyse the relationship between QPRT expression level and survival. Ten datasets (2822 samples) assessed the relationship between QPRT and overall survival. The pooled analysis showed that high QPRT might be associated with shorter overall survival (HR 1.35, 95% CI 1.24 1.47, *P* < 0.00001) (Fig. 2D). Eleven datasets (2085 samples) assessed the relationship between QPRT and recurrence-free survival. The pooled analysis showed that high QPRT might be associated with shorter recurrence-free survival (HR 1.19, 95% CI 1.04 1.35, *P* = 0.009) (Fig. 2E). Twelve datasets (3180 samples) assessed the relationship between QPRT and disease-free survival. The pooled analysis showed that high QPRT might be associated with shorter disease-free survival (HR 1.22, 95% CI 1.07 1.39, *P* = 0.003) (Fig. 2F). Seventeen datasets (4100 samples) assessed the relationship between QPRT and distant metastasis-free survival. The pooled analysis showed that high QPRT might be associated with shorter distant metastasis-free survival (HR 1.25, 95% CI 1.17 1.33, *P* < 0.00001) (Fig. 2G).

### The methylation level of QPRT and prognosis in breast cancer

**Figure 3:**
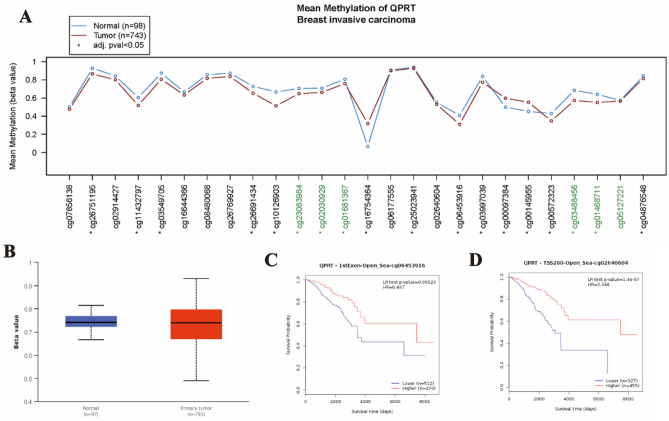
The methylation level of QPRT and prognosis in breast cancer. (**A**) The mean methylation of QPRT in breast cancer showed from the MethSurv database. (**B**) The promotor expression levels of QPRT between primary tumor and normal breast cancer tissues in TCGA were analyzed by the UALCAN database. (**C**) The relationship between QPRT methylation level and OS (cg06453916). (**D**) The relationship between QPRT methylation level and OS (cg02640602).

Based on TCGA data, the QPRT promoter methylation level was higher in breast cancer tissue than in normal breast tissue (*P* < 0.01) (Fig. 3B). To explore the correlation between the QPRT methylation level and expression in breast cancer, 26 methylation sites were obtained with CpG islands highlighted in green. At three sites, cg16754364, cg00097384, and cg00145955, hypermethylation, compared to the methylation level in normal breast tissue, was observed in tumour tissue and at the other 15 sites, the methylation sites tended to be highly methylated in normal breast tissue rather than in tumour tissue, which meant that they had a poor resolution in detecting hypermethylation in tumours (Fig. 3A). Using Methsurv data, survival analyses showed that two hypermethylation sites of QPRT (cg02640602 and cg06453916) correlated with better prognoses in OS (*P* < 0.01) (Fig. 3C–D).

### The predictive values of QPRT in breast cancer treatments

To validate predictive biomarkers in breast cancer, we assessed the relationship between QPRT expression and treatment response. Among those patients who received taxane (AUC = 0.58, *P* = 1.2e−02) or anthracycline (AUC = 0.58, *P* = 4.6e−03), QPRT expression was associated with worse relapse-free survival at 5 years. Among the patients who were administered taxane (AUC = 0.54, *P* = 1.1e−02) or anthracycline (AUC = 0.53, *P* = 3.2e−02), the utilization of QPRT may be indicative of higher rates of pathological complete response (pCR) (Fig. S3). It is worth noting that a higher pCR rate is associated with a poorer prognosis. For patients receiving other therapies (including tamoxifen, an aromatase inhibitor, trastuzumab, lapatinib, ixabepilone, etc.), QPRT might not predict the survival benefits (*P* > 0.5).

### The expression of QPRT and acquired drug resistance

**Figure 4:**
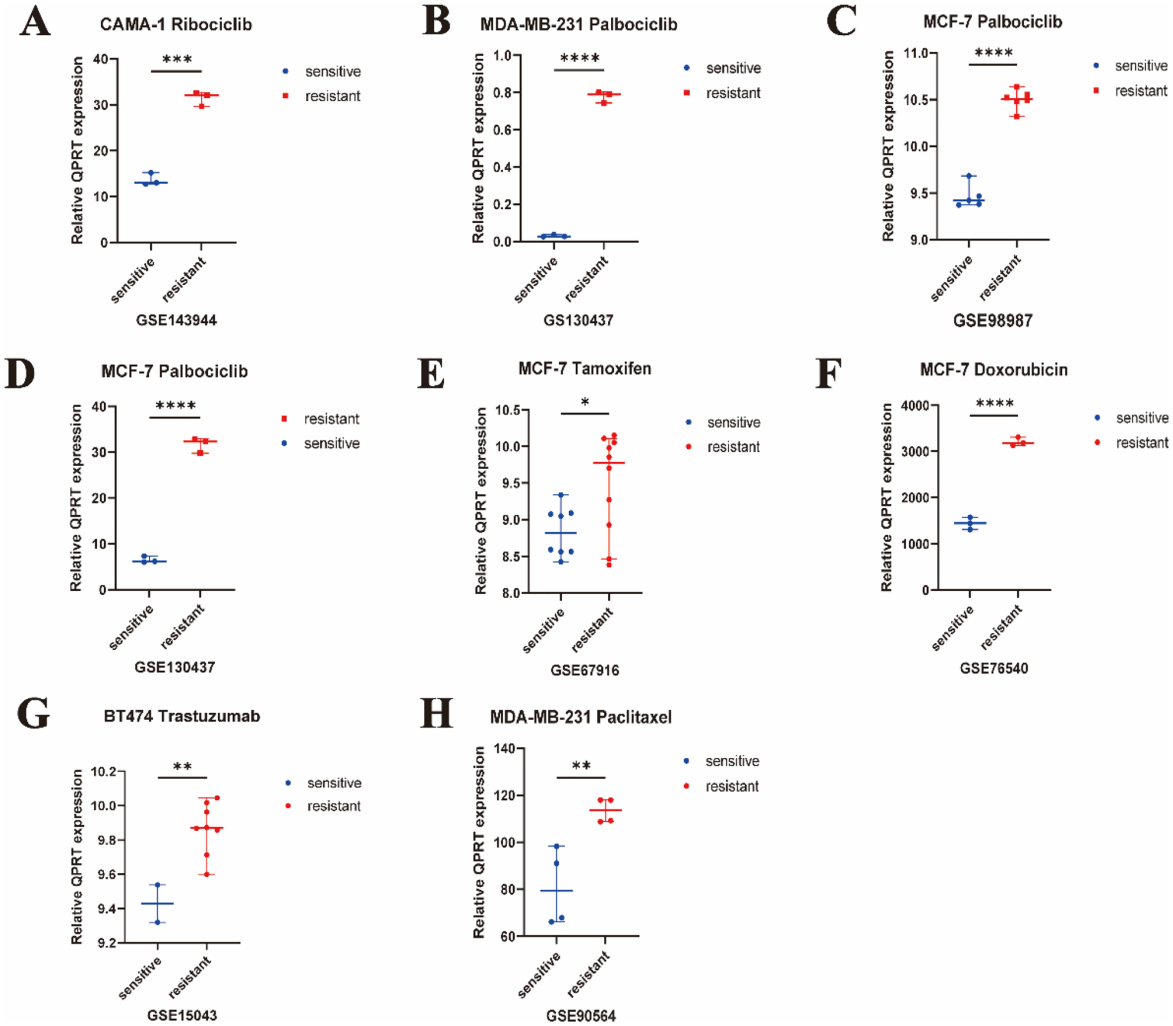
The expression of QPRT between acquired drug-resistant and sensitive cell lines. (**A**) The expression of QPRT between Ribociclib-resistant and sensitive CAMA-1 cell line in GSE143944. (**B**) The expression of QPRT between Palbociclib-resistant and sensitive MDA-MB-231 cell line in GSE130437. (**C**) The expression of QPRT between Palbociclib-resistant and sensitive MCF-7 cell line in GSE98987. (**D**) The expression of QPRT between Palbociclib-resistant and sensitive MCF-7 cell line in GSE130437. (**E**) The expression of QPRT between Tamoxifen-resistant and sensitive MCF-7 cell line in GSE67916. (**F**) The expression of QPRT between Doxorubicin-resistant and sensitive MCF-7 cell line in GSE76540. (**G**) The expression of QPRT between Trastuzumab-resistant and sensitive BT474 cell line in GSE15043. (H) The expression of QPRT between Paclitaxel-resistant and sensitive MDA-MB-231 cell line in GSE90564. Two-tailed unpaired Student’s t-test was used to calculate *P* values. (**A**)–(**D**) Data are presented as the median ± 95% CI. *P < 0.05**P < 0.01*** *P* value < 0.001, **** *P* < 0.0001.

In terms of CDK4/6 inhibitors, QPRT might be a resistant biomarker for ribociclib (CAMA-1, GSE143944, *P* = 0.0001) (Fig. 4A) and palbociclib (MDA-MB-231, GSE130437, *P* < 0.0001 & MCF 7, GSE98987, *P* < 0.0001 & MCF-7, GSE130437, *P* < 0.0001) (Fig. 4B–D). QPRT might be a resistant biomarker for tamoxifen (MCF 7, GSE67916, *P* = 2.5e−02) (Fig. 4E), doxorubicin (MCF 7, GSE76540, *P* = 1.9e−03) (Fig. 4F), trastuzumab (BT474, GSE15043, *P* = 6.3e−03) (Fig. 4G), and paclitaxel (MDA-MB-231, GSE90564, *P* = 3.4e−02) resistance (Fig. 4H).

### The related genes and function prediction results of QPRT

We obtained 685 genes significantly correlated with QPRT from LinkedOmics database. Among those genes, 368 genes were positively correlated (r > 0.25, *P* < 0.05), and 317 genes were negatively correlated (r < − 0.25, *P* < 0.05).

To elucidate the biological functions and pathways associated with QPRT, GO enrichment and KEGG pathway analyses were performed, and the results showed that related biological processes included regulation of the G1/S transition of the mitotic cell cycle, protein ubiquitination, regulation of the G0–G1 transition, positive regulation of the Wnt signalling pathway and positive regulation of B-cell proliferation (Fig. S4A). For molecular function, QPRT was associated with protein binding, siRNA binding, and glutathione hydrolase activity (Fig. S4B). For cellular components, QPRT was associated with the nucleus, cytoplasm, cytosol, nucleoplasm, and extracellular exosome (Fig. S4C). Furthermore, KEGG pathway analysis indicated that RNA Polymerase II Transcription, generic transcription pathway, TCR signalling, and the ER‒phagosome pathway were also enriched (Fig. S4D).

**Figure 5:**
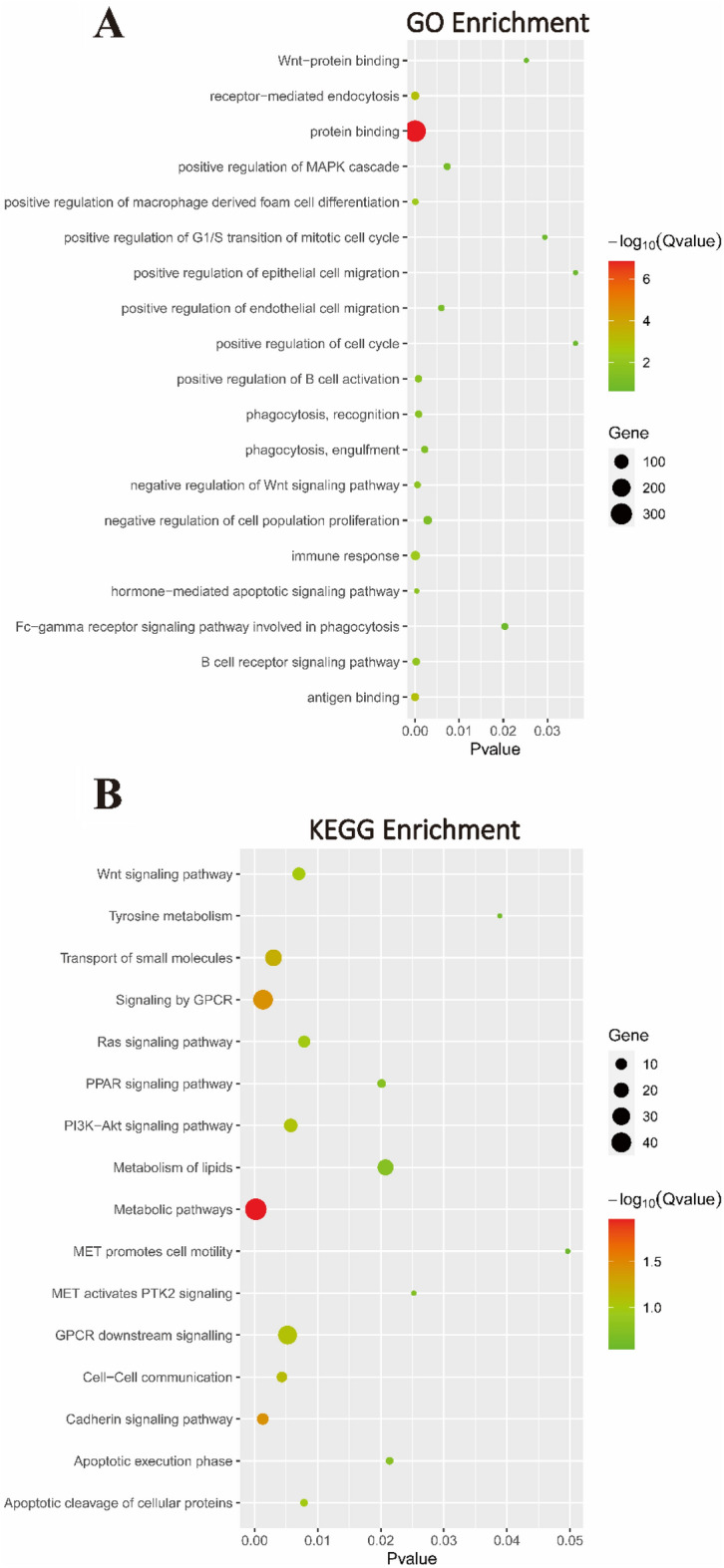
The function prediction results of QPRT in breast cancer. (**A**) Significantly enriched GO terms and (**B**) top enriched KEGG pathways were showed by analyzing GEO dataset (GSE 151521).

By analysing the GEO dataset GSE151521, we identified several QPRT knockdown related genes and conducted enrichment analysis. We found that the knockdown of QPRT significantly enriched terms related to the Wnt signalling pathway, MAPK signalling pathway, cell cycle, and cell proliferation in GO enrichment analysis. Additionally, macrophage-related enrichment in the immune response occurred frequently (Fig. 5A). In KEGG enrichment analysis, we observed enrichment in pathways such as the Wnt signalling pathway, PPAR signalling pathway, PI3K-AKT signalling pathway, and apoptosis (Fig. 5B). Both QPRT-related genes in the LinkedOmics database and genes associated with QPRT knockdown showed correlations with the Wnt signalling pathway and cell cycle. We speculate that QPRT may play a role in breast cancer through the regulation of the cell cycle and modulation of molecules in the Wnt signalling pathway.

### The relationship between QPRT single-cell analyses and immune infiltrate abundances in breast cancer

**Figure 6:**
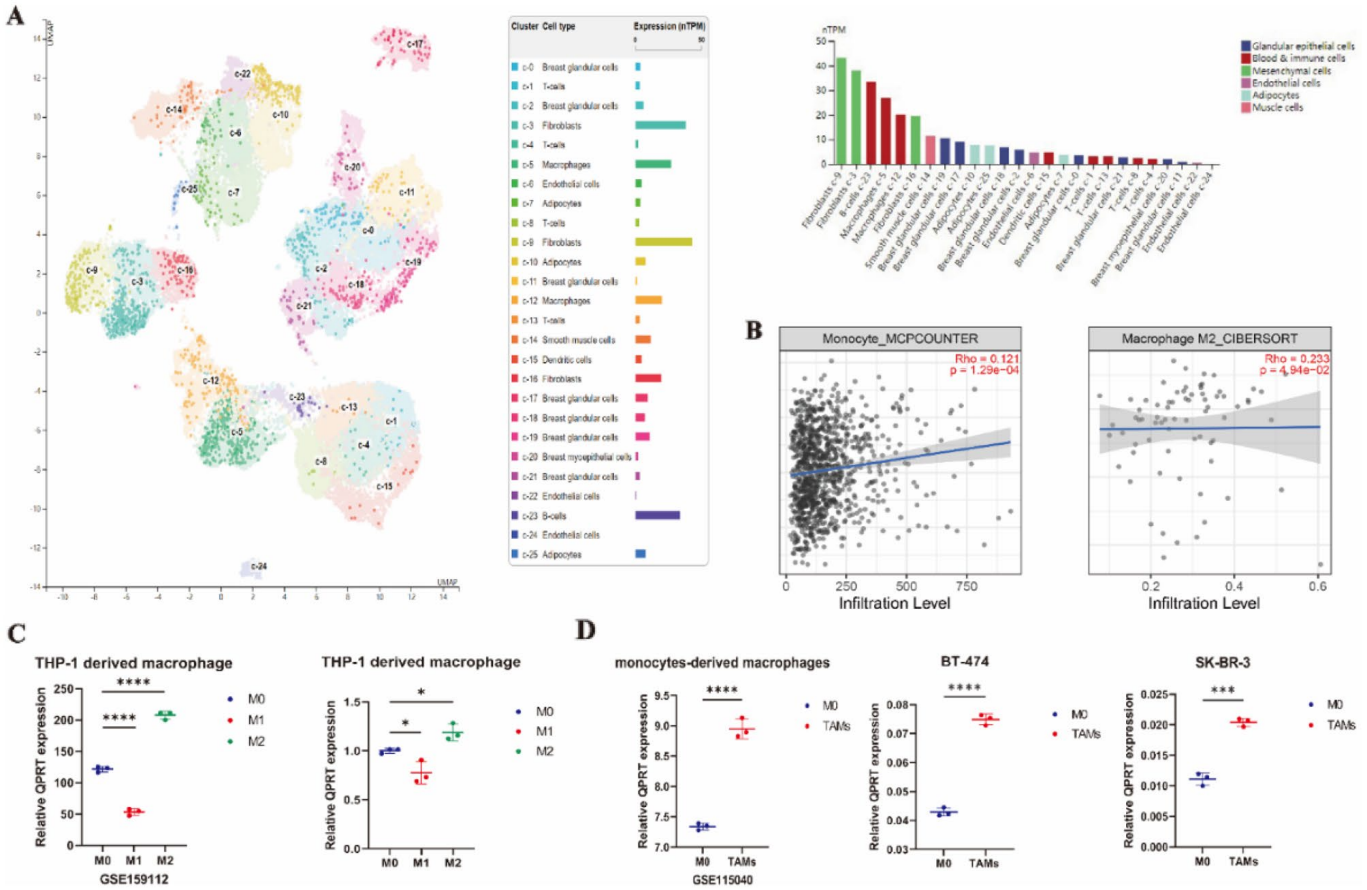
The relationship between QPRT single-cell analyses and immune infiltrates' abundances in breast cancer. (**A**) The relationship between QPRT expression and different single-cell types in breast tissues. (**B**) The relationship between QPRT and different macrophage cell infiltration. (**C**) M0 macrophages: PMA-stimulated THP-1 cells for 48 h (PMA 100 ng/ml), M1 macrophages: LPS (100 ng/ml) and IFN-γ(20 ng/ml) induce M0 macrophages to M1-like phenotype, M2 macrophages: IL-4(20 ng/ml) and IL-13(20 ng/ml) induce M0 macrophages to M2-like phenotype, TAMs (tumor-associated macrophages): culture supernatant of SK-BR-3 cells or BT-474 cells were added in M0 macrophages in proportion co-culture for 48 h. The scatter plot shows the expression of QPRT among M0 macrophages, M2 macrophages, and TAMs. Two-tailed unpaired Student’s t-test was used to calculate *P* values. (**D**) The expression of QPRT among M0 macrophages, M2 macrophages, and TAMs in GEO. (**C**,**D**) Data are presented as the mean ± 95% CI. **P < 0.01, ***P < 0.001, ****P < 0.0001.

Based on the human protein atlas data, breast tissue could be divided into 25 clusters by single-cell sequencing analysis. In these 25 clusters, QPRT was mainly highly expressed in fibroblasts, B cells, and macrophages (Fig. 6A). Furthermore, single-cell sequencing of other tissues found that QPRT was highly expressed in macrophages (Fig. S5). This suggested that QPRT may affect the microenvironment, through macrophages, to promote or inhibit the occurrence of disease.

To visualize the correlation between QPRT expression and immune infiltration levels in breast cancer, a large number of available TCGA samples were used. QPRT was positively associated with monocyte cell (r = 0.12, *P* = 1.3e−04), M2 macrophage cell (r = 0.23, *P* = 4.9e−02) (Fig. 6B), central memory CD4+ T-cell (r = 0.38, *P* = 2.1e−03), B-cell (r = 0.19, *P* = 7.5e−06), NK cell (r = 0.16, *P* = 2.3e−02), neutrophil cell (r = 0.15, *P* = 4.6e−02), myeloid dendritic cell (r = 0.15, *P* = 7.0e−04), T-cell of NK (r = 0.15, *P* = 8.4e−04), follicular helper T-cell (r = 0.12, *P* = 5.5e−03), MDSC (myeloid-derived suppressor cells) (r = 0.12, *P* = 7.6e−03) and regulatory T-cell (r = 0.11, *P* = 1.5e−02) infiltration but negatively associated with mast cell (r = − 0.17, *P* = 1.9e−02), CD8+ T-cell (r = − 0.36, *P* = 1.9e−03), haematopoietic stem cell (r = − 0.11, *P* = 4.6e−04), common lymphoid progenitor (r = − 0.18, *P* = 1.4e−02) and granulocyte-monocyte progenitor (r = − 0.16, *P* = 3.3e−02) (Fig. S6).

Moreover, the expression of QPRT in HER2+ breast cancer is significantly higher than that in other subtypes (Fig. 1B), so we chose to explore the relationship between QPRT and macrophages in HER2+ breast cancer. We found that higher expression of QPRT was found in M2 macrophages than in M0 macrophages by qPCR, and the same results were validated in the GSE159112 database (Fig. 6C). Furthermore, high QPRT expression was also found in TAMs (M0 macrophages cocultured with SK-BR-3 cell or BT-474 cell supernatants) compared with M0 macrophages in HER2+ breast cancer. Similar expression changes were also observed in the GSE115040 datasets (Fig. 6D).

### The correlation of QPRT expression with biomolecules and comparative toxicogenomics

**Figure 7:**
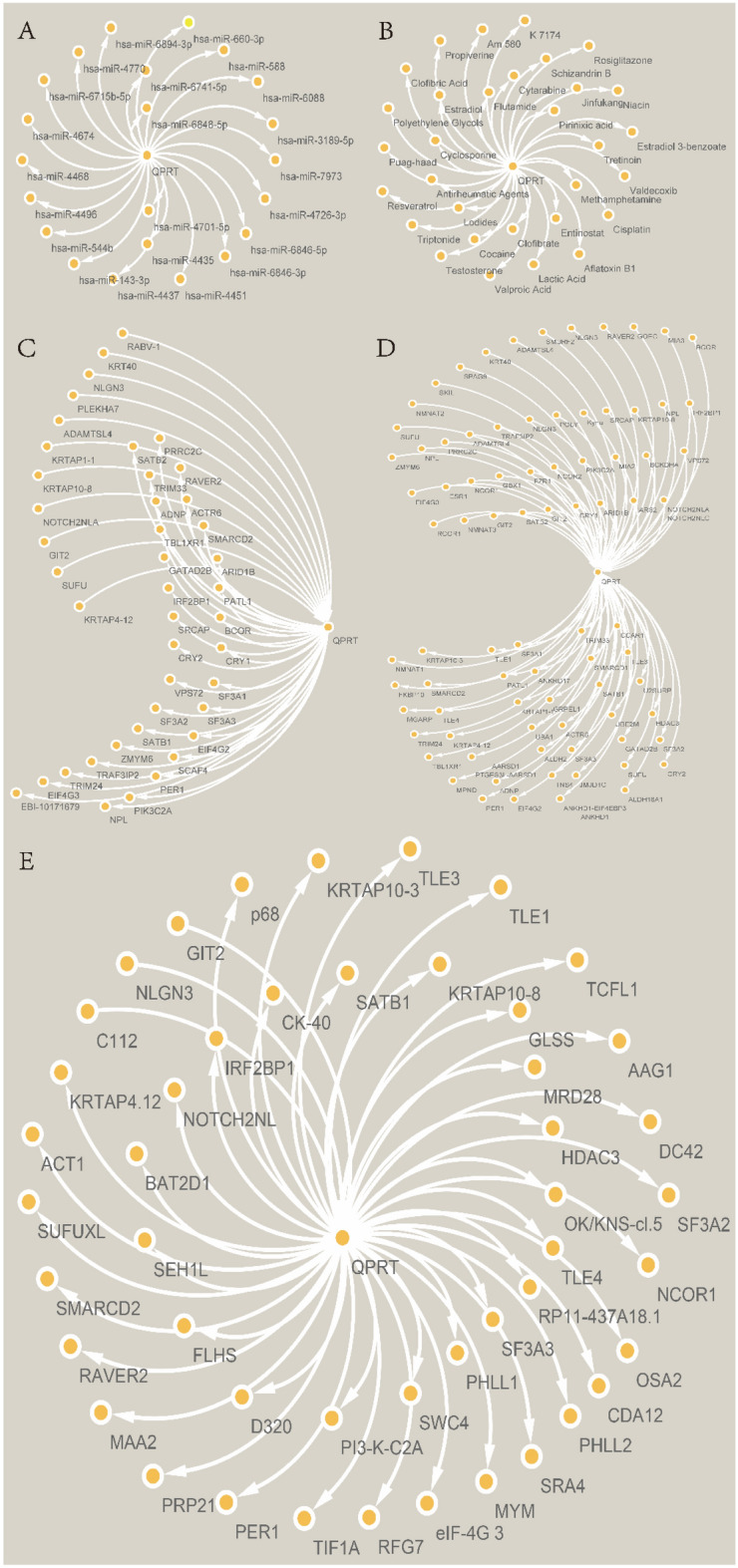
The correlation of QPRT expression with biomolecules and comparative toxicogenomics. (**A**) The miRNAs that might be associated with QPRT. (**B**) The comparative toxicogenomics that might be associated with QPRT. (**C**) QPRT was searched in the IMEx database and the corresponding protein–protein interaction network was obtained. (**D**) QPRT was searched in the iRefIndex database and the corresponding protein–protein interaction network was obtained. (**E**) QPRT was searched in the BioGrid database and the corresponding protein–protein interaction network was obtained.

To predict miRNA binding sites related to QPRT, we explored the TargetScan database and found that 612 miRNAs might regulate QPRT. The most likely targets in these sites were hsa-miR-4763-5p, hsa-miR-7973, hsa-miR-4674, hsa-miR-4437, hsa-miR-4468, hsa-miR-4701-5p and hsa-miR-3189-5p (context ++ score < − 0.5 and context ++ score percentile > 99) (Fig. 7A). To further explore possible drug targets of QPRT, the Comparative Toxicogenomics Database (CTD) was used. The results visualized by Cytoscape showed that a series of drugs can target QPRT, such as antineoplastic drugs such as cisplatin, Jinfukang, flutamide, and entinostat and substances in the human body such as oestradiol, lactic acid, and testosterone (Fig. 7B). The intersection of the above results and the results of the CMAP website analysis found that these drugs appeared repeatedly, including clofibrate, cytarabine, entinostat, estradiol, flutamide, niacin, resveratrol, rosiglitazone, testosterone, tretinoin and valdecoxib. This suggests that QPRT is more likely to be associated with these drugs and affect breast cancer directly or indirectly. In addition, based on the three databases of iRefIndex, BioGrid and IMEx, we draw the protein–protein interaction networks (PPI) related to QPRT respectively (Fig. 7C–E). We summarized the results of these three databases and found that these genes or proteins appeared in these three databases, including PKLR, AMBP, APOC3, RBP4, APOM, TF, UBE2J1, DNAJB9, SEC61B, HILPDA, LIPA, YIPF5, SEC23IP and SEC24C. We reasonably believe that QPRT is likely to interact with these genes or proteins and affect the progression of breast cancer. We speculated that these biomolecules and comparative toxicogenomics can prevent breast cancer deterioration and even improve patient survival status.

## Discussion

QPRT is the key enzyme in the catabolism of quinolinic acid, which is known as an intermediate in the kynurenine pathway of tryptophan to nicotinamide adenine dinucleotide (NAD). Regarding this tryptophan metabolic pathway, many studies have already shown that tryptophan metabolism plays an important role in cancer progression by inhibiting the antitumour immune response and promoting the malignant characteristics of cancer cells. It was reported that degrading enzymes in the kynurenine pathway were expressed in various types of cancer and related to adverse clinical outcomes^29^. There is evidence showing that NAD+ exerts a role in tumour biology through tryptophan and that inhibition of de novo NAD+ synthesis promotes tumourigenesis through DNA damage in the liver^30^. Additionally, some research has shown that the metabolite of tryptophan can promote the movement and metastasis of cancer cells in glioblastoma^31^ and breast cancer^32^. Moreover, accumulation of endogenous tryptophan-derived metabolites^33^, immune-related effects^34^, and expression status^35^ had influences on breast cancer, although kynurenic acid had not been previously associated with breast cancer risk^36^. The potential mechanisms of this metabolic pathway mediating disease include tryptophan-dependent protein toxicity, excitatory toxicity caused by the accumulation of tryptophan metabolites, and energy imbalance caused by NAD+ depletion, containing local restrictive changes in downstream metabolites in the tumour microenvironment^37^. Based on the above results, we believe that QPRT is potentially very important in the genesis, progression, metastasis, and invasion of breast cancer.

Liu et al. found that QPRT expression levels were upregulated in breast cancer and that knockdown of QPRT could inhibit breast cancer cell migration and invasion^17^. Upregulated QPRT promoted growth, migration, and invasion and increased drug resistance^10^. Similarly, we found that the expression level of QPRT in breast cancer tissues was significantly higher than that in normal tissues. The UALCAN and bc-GenExMiner online tools revealed that HER2 positivity, nodal metastasis status, and TP53 mutation were positively correlated with high QPRT expression. We further investigated the prognostic value of QPRT in breast cancer using the Kaplan–Meier Plotter and bc-GenExMiner databases. Patients with high QPRT expression levels had worse survival prognosis in OS as well as DMFS and RFS, either ER+, PR+, and HER2+ receptor status or molecular subtyping. In ER+ breast cancer, DSCAM-AS1 increased QPRT expression and caused poor prognosis^11^. These findings collectively demonstrated that QPRT might be a prognostic biomarker for breast cancer.

A previous study showed that overexpression of QPRT increased partial resistance to imatinib in K562 cells, suggesting that QPRT may have an anti-apoptotic function^10^. By using the ROC plotter online analysis tool, we demonstrated that taxane and anthracycline, as predictive biomarkers in breast cancer, were statistically significant with the area under the curve (AUC). Furthermore, we analysed GEO datasets and found that QPRT was able to be a biomarker for taxane, trastuzumab, paclitaxel, doxorubicin, and tamoxifen via acquired drug resistance. Coincidentally, we found that there was an obvious high relevance between QPRT and antineoplastic drugs such as cisplatin, Jinfukang, and entinostat by using the comparative toxicogenomics database (CTD) analysis tool. These findings indicate that QPRT may act as a potential target and biomarker to indicate drug resistance and the clinical efficacy of breast cancer antitumour drugs.

In our enrichment analysis of QPRT, a significant association with the cell cycle was observed. QPRT is capable of regulating the transition from G0 to G1 and influencing the G1/S transition of the mitotic cell cycle. By analyzing the GEO dataset, it was found that the expression of QPRT in the CDK4 / 6 inhibitor palbociclib resistance group was higher than that in the control group. Interestingly, we also identified a certain relationship between QPRT and the cell cycle drug cytarabine. It is well known that cytarabine, as a pyrimidine antimetabolite mainly targeting the S phase of cell proliferation in clinical settings, exerts a noticeable anti-tumour effect by inhibiting cell DNA synthesis and impeding cell proliferation^38^. We hypothesize that QPRT is likely to be directly or indirectly involved in cell cycle regulation and consequently impacts the occurrence and progression of breast cancer.

It has been shown that QPRT promotes breast cancer progression by activating the PI3K/Akt pathway^39^. Consistently, our study revealed a significant enrichment of QPRT in the PI3K-AKT signalling pathway. Moreover, QPRT is also associated with the gene-drug effects of resveratrol and estradiol. Resveratrol has been reported to induce apoptosis in cancer cells by downregulating PI3K, leading to cell cycle arrest^40^. On the other hand, chronic exposure to high levels of estradiol has been identified as a major cause of ER-positive breast cancer^41^, and it can upregulate Twist via the PI3K/AKT/NF-κB signalling pathway, thereby promoting the progression of hormone-dependent breast cancer^42^.These findings further support the notion that QPRT can influence the progression of breast cancer through the PI3K-AKT signalling pathway.

Additionally, rosiglitazone, a drug used for diabetes treatment, has shown positive anti-tumour effects in breast cancer. Research has demonstrated that the combination of rosiglitazone with MEK inhibitors can induce the differentiation of breast cancer cells into adipocytes, reducing tumour invasiveness and suppressing tumour metastasis^43^. In our study, we found that QPRT is significantly enriched in the PPAR signalling pathway and exhibits a relationship with rosiglitazone. It is possible that rosiglitazone and QPRT may also have a similar promoting or inhibitory relationship, thereby influencing the occurrence and development of tumours.

Jones et al. found that QPRT expression correlated significantly with neopterin, a pro-inflammatory immune response marker^44^. In follicular thyroid carcinoma, QPRT might be a potential marker in immunohistochemical features^45^. According to our results, QPRT was positively associated with the infiltration of many immune cells, such as central memory CD4+ T cells, regulatory T cells, M2 macrophages, B cells, monocytes, NK cells, and neutrophils, but negatively associated with mast cells and CD8+ T cells. Significantly, our enrichment analysis revealed the presence of various macrophage-related biological processes, including the Fc-gamma receptor signaling pathway associated with phagocytosis. Further investigation indicated that QPRT was more highly expressed in M2 macrophage cells than in M0 macrophage cells, and the expression of QPRT in two different tumour-associated macrophages was also higher than that in M0 macrophages. In terms of tumour growth, M2 macrophages can accelerate cancer cells growth by releasing growth factors^46^. Promoting M2 polarization or increasing the ratio of M2/M1 can promote tumour proliferation and development^47–52^, and M2-polarized macrophages have obvious proangiogenic and protumourigenic effects^53^. In terms of tumour invasion and metastasis, increased M2 macrophages in the tumour immune microenvironment can drive breast cancer metastasis^54^. Tumour-promoting M2 macrophages enriched in the tumour microenvironment increase the invasiveness of triple-negative breast cancer (TNBC)^55^, and CHI3L1 secreted by M2 macrophages promotes the metastasis of gastric cancer and breast cancer cells in vitro and in vivo^56^. In addition, studies have shown that M2 macrophages are associated with poor prognoses in tumours, such as TNBC^57^ and oral squamous cell carcinoma^50^. Immunosuppression is also a known effect of M2 macrophages, which can damage the antitumour immunity of the tumour microenvironment^58–60^. In tumour treatment resistance and drug resistance, M2 macrophages can not only promote the chemotherapy resistance of cancer cells^61^ but also mediate the radiation resistance of inflammatory breast cancer (IBC) cells with cocultured IBC cell lines^62^. Studies have shown that QPRT overexpression activates the phagocytosis of macrophages and vice versa. Inhibition of QPRT increased the surface markers of M1 macrophages and decreased the surface markers of M2 macrophages^63^. We reasonably suppose that QPRT may play a tumour-promoting role through M2 macrophages. Although in GSE55015^64^, QPRT had meaningless miRNA and mRNA expression of in vitro tumour-educated macrophages (TEM), the identified miRNAs through in vitro profiling of TEM had longer DFS in ER+ breast cancers. The results showed that under the effect of QPRT, breast cancer could be regulated by immune cells in development and prognosis.

QPRT has been reported, but only in a few types of cancers. Multiple genes or proteins interact with QPRT in different ways and participate in the occurrence, development, invasion, and metastasis of breast cancer, but few have been verified. It is essential to predict the function and related molecules of QPRT. Identifying the function of QPRT will not only elucidate the role of the gene but will also provide ideas for potential therapeutic targets for research on new drugs and antitumour drug resistance.

## Conclusion

QPRT may be a new target for breast cancer for both cancer cells and tumour-educated macrophages. QPRT was higher in breast cancer tissue, and its higher level represented worse survival. The worse survival of high QPRT might be due to acquired drug resistance to, for example, chemotherapy, trastuzumab, endocrine drugs, and CDK4/6 inhibitors. Meanwhile, QPRT might also activate the PI3K-AKT and cell cycle to accelerate tumour growth and survival. QPRT might be a biomarker for TAMs and M2 macrophages, and QPRT might function via the Fc-epsilon receptor signalling pathway in macrophages.

### Supplementary Information

Supplementary Figures.

## Data Availability

The data used to support the findings of this study are included in the article.
