# Supplementary material for: A comprehensive analysis of the role of QPRT in breast cancer
Source: Sci Rep. 2023 Sep 18;13:15414. doi: 10.1038/s41598-023-42566-4 (PMC10507026; doi:10.1038/s41598-023-42566-4)
Supplement: Supplementary file 1 — Supplementary Figures. [file 41598_2023_42566_MOESM1_ESM.docx]

**­­Supplementary Materials**

| 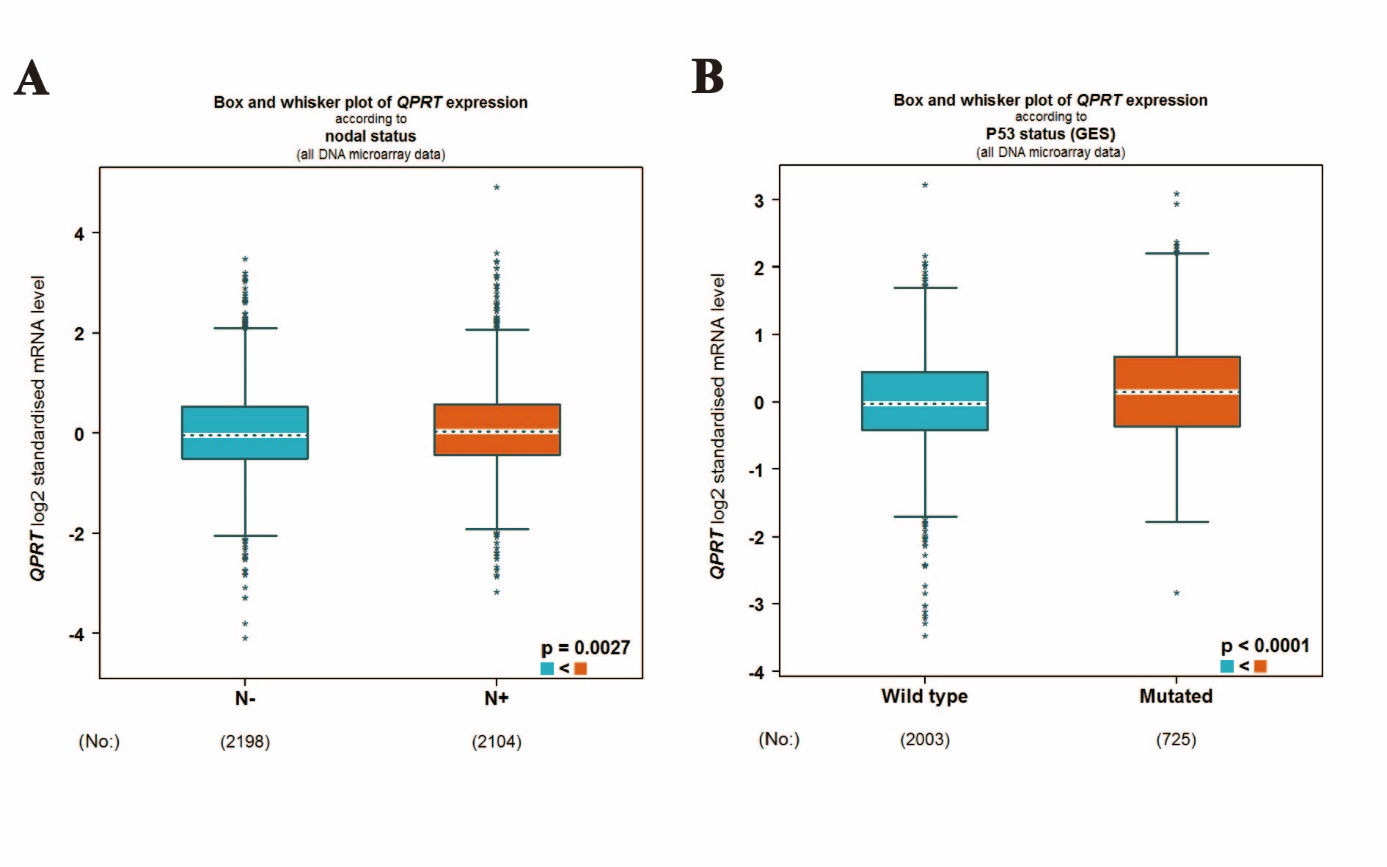 |
| --- |

**Figure S1: The expression and characteristics of QPRT in breast cancer.**

(A) The expression of QPRT in GEO between different node statuses in breast cancer using the bc-GenExMiner database. (B) The expression of QPRT in GEO between wild type and mutated P53 statuses in breast cancer using the bc-GenExMiner database.

| 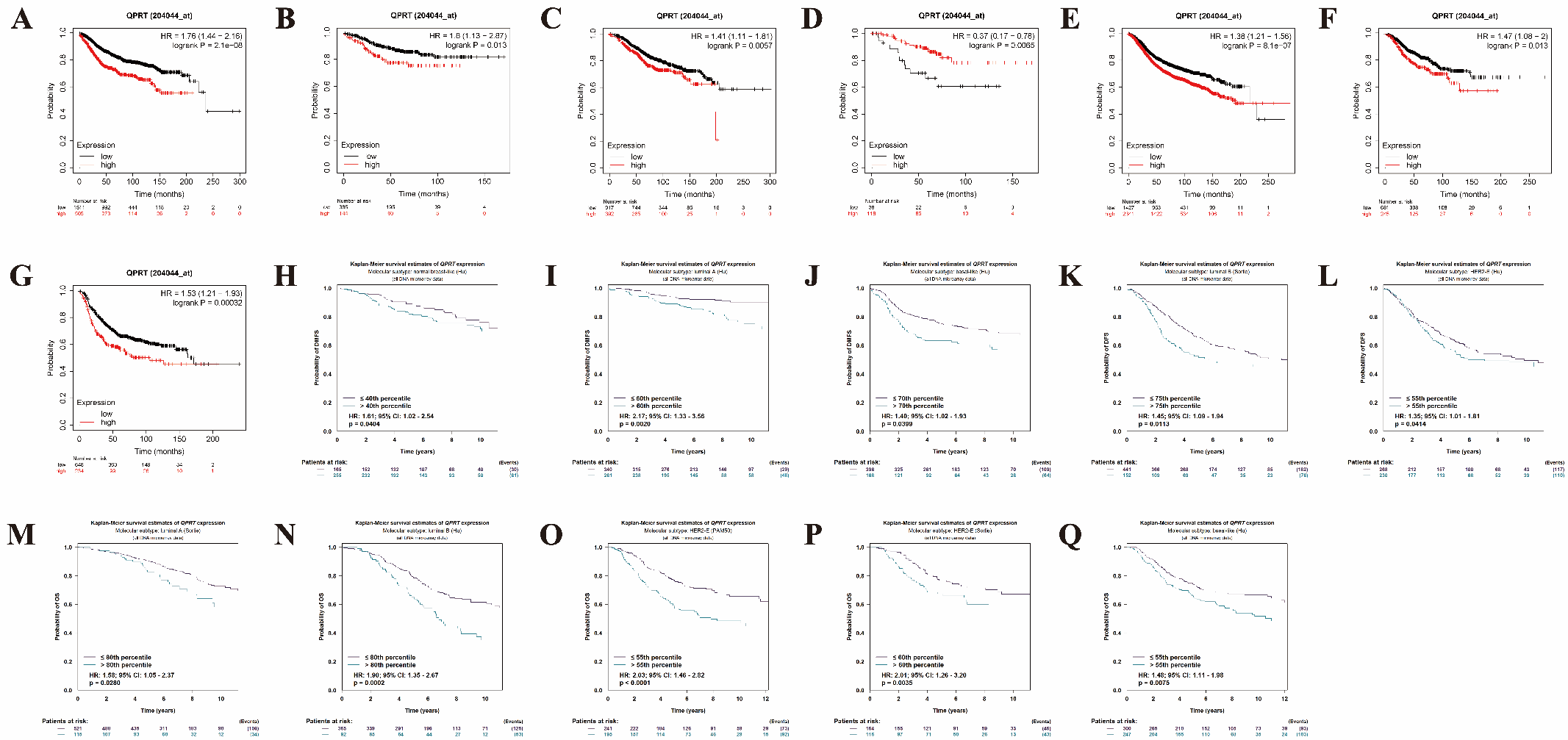 |
| --- |

**Figure S2: The relationship between QPRT expression and prognosis values in breast cancer.**

(A) The relationship of QPRT expression levels and distant metastasis-free survival (DMFS) in ER+ breast cancer. (B) The relationship of QPRT expression levels and DMFS in PR+ breast cancer. (C) The relationship of QPRT expression levels and overall survival (OS) in ER+ breast cancer. (D) The relationship of QPRT expression levels and OS in PR+ breast cancer. (E) The relationship of QPRT expression levels and relapse-free survival (RFS) in ER+ breast cancer. (F) The relationship of QPRT expression levels and RFS in PR+ breast cancer. (G) The relationship of QPRT expression levels and RFS in HER2+ breast cancer. (H) The relationship of QPRT expression levels and DMFS in normal breast-like breast cancer (Hu). (I) The relationship of QPRT expression levels and DMFS in luminal A breast cancer (Hu). (J) The relationship of QPRT expression levels and DMFS in basal-like breast cancer (Hu). (K) The relationship of QPRT expression levels and RFS in luminal B breast cancer (Sorile). (L) The relationship of QPRT expression levels and RFS in HER2-E breast cancer (Hu). (M) The relationship of QPRT expression levels and OS in luminal A breast cancer (Sorile). (N) The relationship of QPRT expression levels and OS in luminal B breast cancer (Hu). (O) The relationship of QPRT expression levels and OS in HER2-E breast cancer (PAM50). (P) The relationship of QPRT expression levels and OS in HER2-E breast cancer (Sorile). (Q) The relationship of QPRT expression levels and OS in basal-like breast cancer (Hu).

| 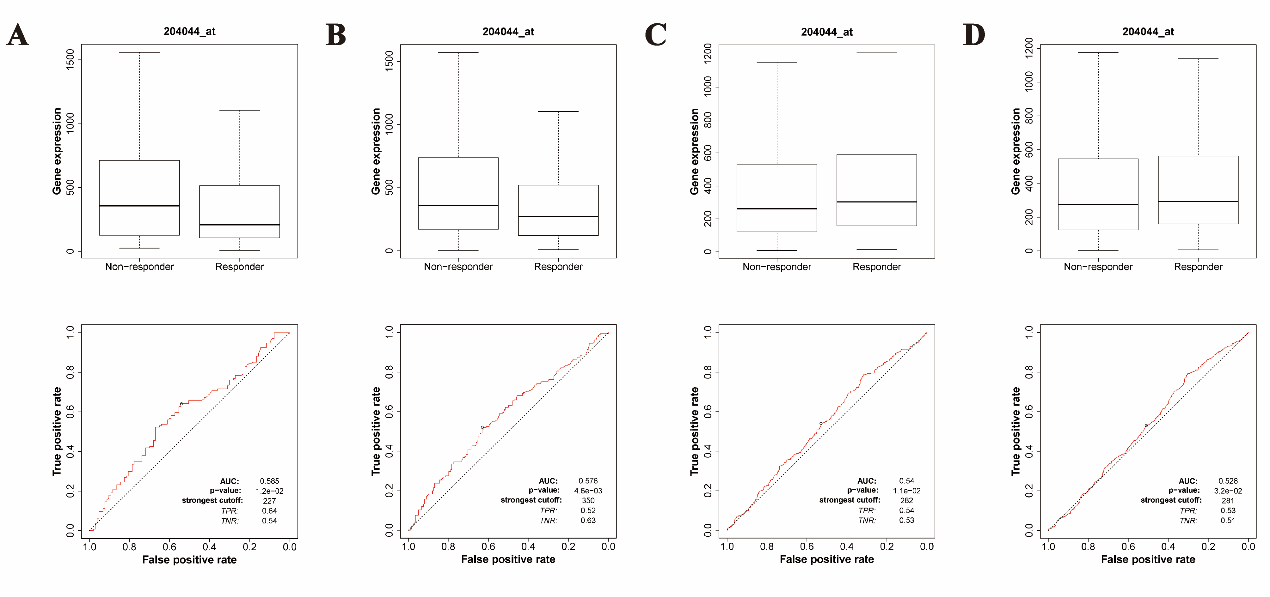 |
| --- |

**Figure S3: The predictive values of QPRT in breast cancer treatments using the ROCplot database.**

(A) The predictive values of QPRT for taxane response in relapse-free survival at 5 years. (B) The predictive values of QPRT for anthracycline response in relapse-free survival at 5 years. (C) The predictive values of QPRT for taxane in pCR. (D) The predictive values of QPRT for anthracycline in pCR.

| 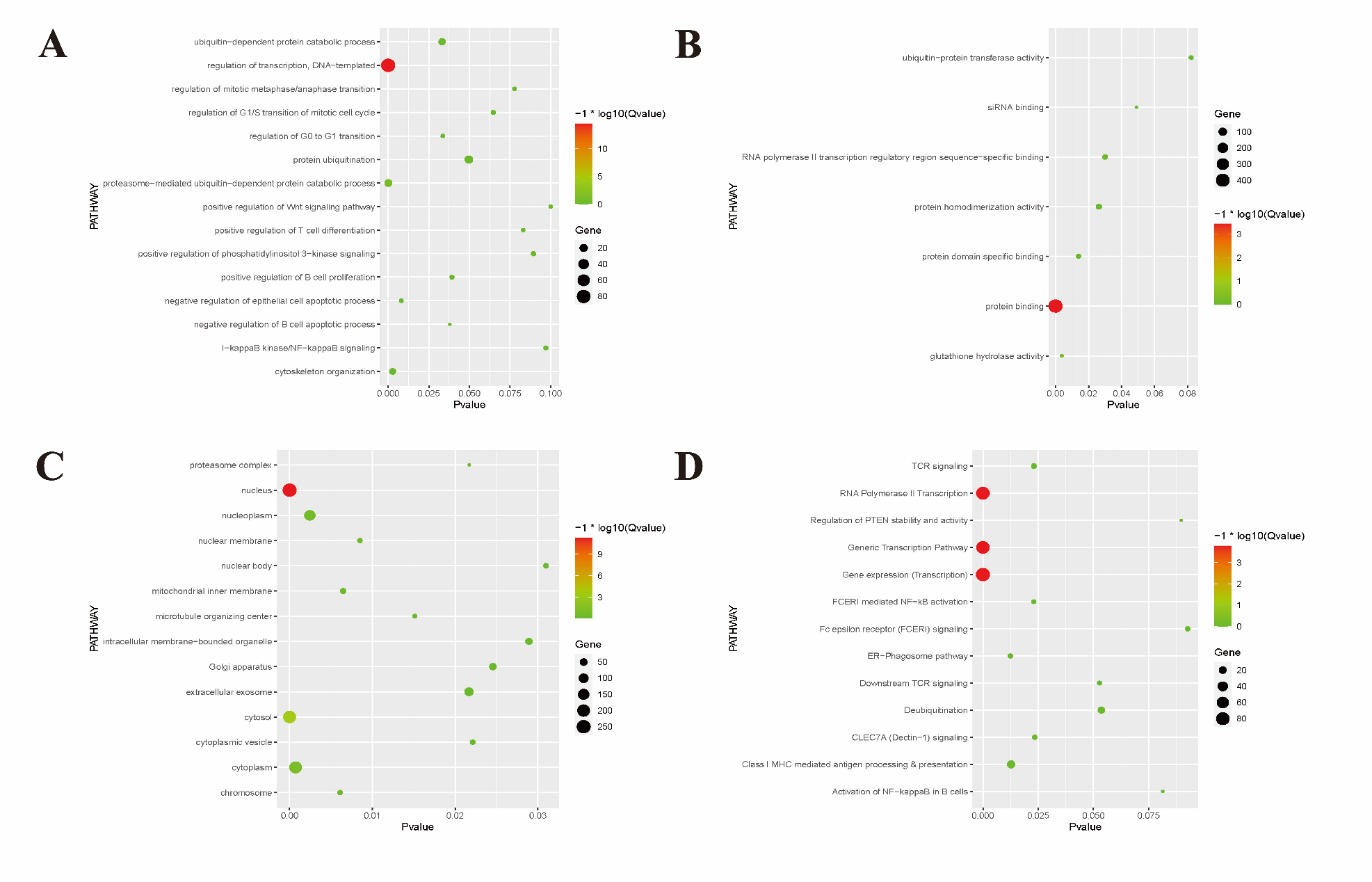 |
| --- |

**Figure S4: The function prediction results of QPRT in breast cancer.**

Through the linkedoimcs database, we screened genes related to QPRT and performed GO and KEGG enrichment analysis on them, including biological process (A), molecular function (B), cellular component(C) and KEGG pathway(D).

| 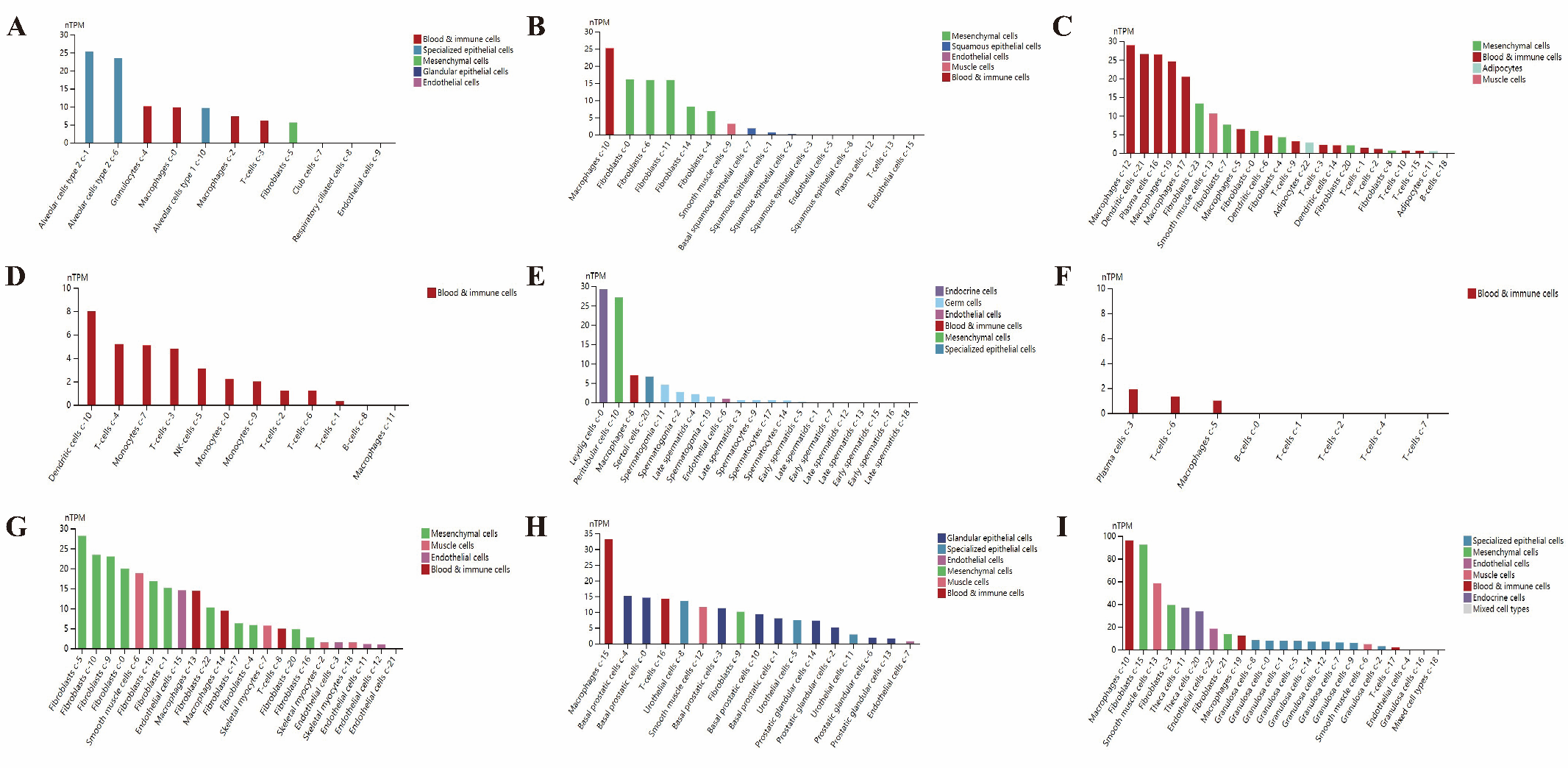 |
| --- |

**Figure S5: QPRT single-cell analyses in different tissues derived from the Human Protein Atlas database.**

(A) The relationship between QPRT expression and different single cell types in lung tissues. (B) The relationship between QPRT expression and different single cell types in esophagus tissues. (C) The relationship between QPRT expression and different single cell types in adipose tissues. (D) The relationship between QPRT expression and different single cell types in peripheral blood mononuclear cells (PBMC). (E) The relationship between QPRT expression and different single cell types in testis tissues. (F) The relationship between QPRT expression and different single cell types in spleen tissues. (G) The relationship between QPRT expression and different single cell types in skeletal muscle tissues. (H) The relationship between QPRT expression and different single cell types in prostate tissues. (I) The relationship between QPRT expression and different single-cell types in ovary tissues.

| 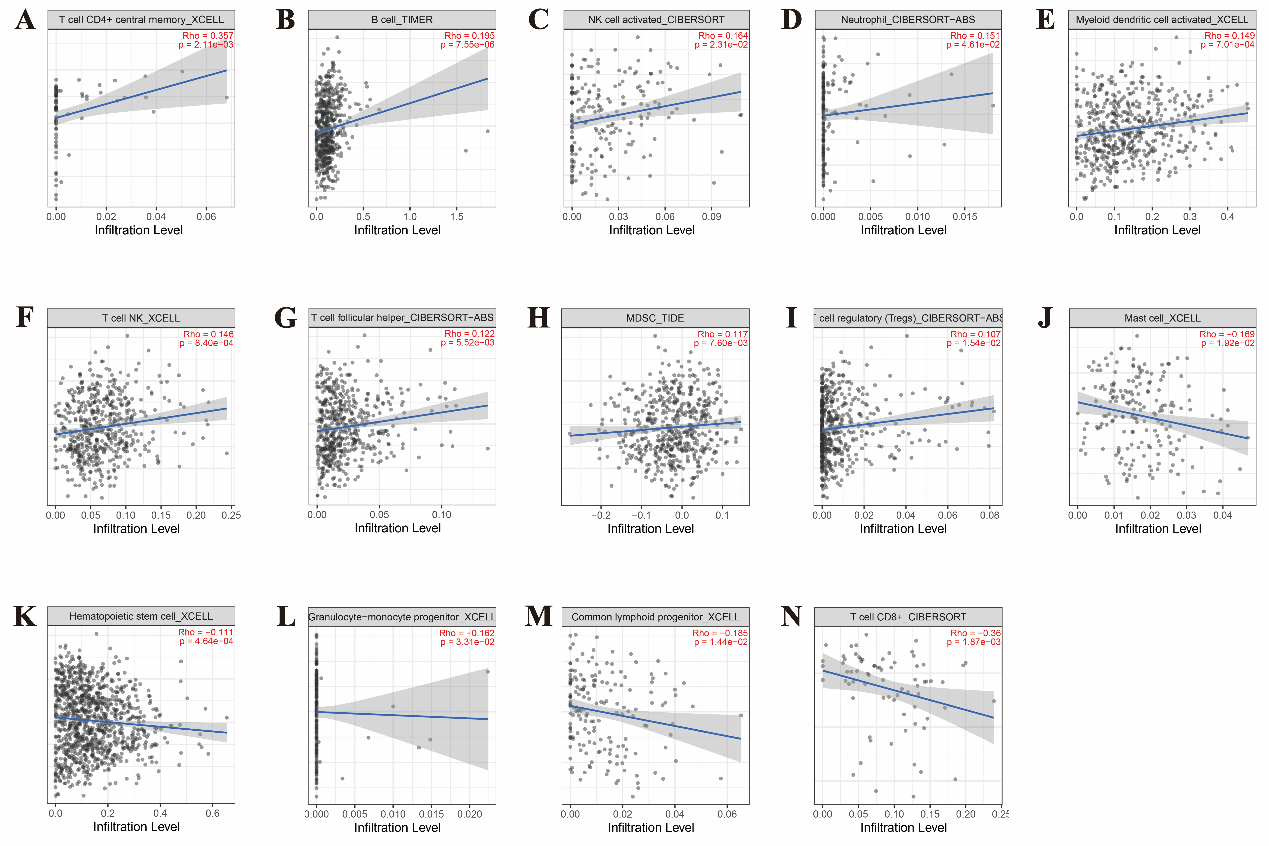 |
| --- |

**Figure S6: The relationship between QPRT and immune infiltrates' abundances in breast cancer shown showed from TIMER2.0.**

(A) The relationship between QPRT and central memory CD4+T cell infiltration. (B) The relationship between QPRT and B cell infiltration. (C) The relationship between QPRT and NK cell infiltration. (D) The relationship between QPRT and neutrophil cell infiltration. (E) The relationship between QPRT and myeloid dendritic cell infiltration. (F) The relationship between QPRT and NK T cell infiltration. (G) The relationship between QPRT and follicular helper T cell infiltration. (H) The relationship between QPRT and myeloid-derived suppressor cells (MDSC) infiltration. (I) The relationship between QPRT and regulatory T cell infiltration. (J) The relationship between QPRT and mast cell infiltration. (K) The relationship between QPRT and hematopoietic stem cell infiltration. (L) The relationship between QPRT and granulocyte-monocyte progenitor infiltration. (M) The relationship between QPRT and common lymphoid progenitor infiltration. (N) The relationship between QPRT and CD8+T cell infiltration.
